# BMI and Waist Circumference; Cross-Sectional and Prospective Associations with Blood Pressure and Cholesterol in 12-Year-Olds

**DOI:** 10.1371/journal.pone.0051801

**Published:** 2012-12-14

**Authors:** Marga B. M. Bekkers, Bert Brunekreef, Gerard H. Koppelman, Marjan Kerkhof, Johan C. de Jongste, Henriëtte A. Smit, Alet H. Wijga

**Affiliations:** 1 Institute for Risk Assessment Sciences, Utrecht University, Utrecht, The Netherlands; 2 Centre for Prevention and Health Services Research, National Institute for Public Health and the Environment (RIVM), Bilthoven, The Netherlands; 3 Julius Centre for Health Sciences and Primary Care, University Medical Center Utrecht, Utrecht, The Netherlands; 4 Department of Pediatric Pulmonology and Pediatric Allergology, Beatrix Children’s Hospital, University of Groningen, University Medical Center Groningen, Groningen, The Netherlands; 5 Department of Epidemiology and Bioinformatics, University of Groningen, University Medical Center Groningen, Groningen, The Netherlands; 6 Department of Pediatrics, Erasmus University Medical Center, Sophia Children’s Hospital, Rotterdam, The Netherlands; Innsbruck Medical University, Austria

## Abstract

**Objective:**

Childhood and adolescent overweight, defined by body mass index (BMI) are associated with an increased risk of cardiovascular disease in later life. Abdominal adiposity may be more important in associations with cardiovascular diseases but waist circumference (WC) has been rarely studied in children. We studied associations between BMI and WC and blood pressure (BP) and cholesterol in 12-year-old children and prospectively changes in BMI or WC status between age 8 and 12 years and BP and cholesterol at age 12.

**Study Design:**

Weight, height, WC, BP and cholesterol concentrations were measured in 1432 children at age 12 years. Linear regression was used to study the associations between high BMI and large WC (>90^th^ percentile) and BP and cholesterol.

**Results:**

Systolic BP was 4.9 mmHg higher (95% (CI 2.5, 7.2) in girls and 4.2 mmHg (95%CI 1.9, 6.5) in boys with a high BMI. Large WC was also associated with higher systolic BP in girls (3.7 mmHg (95%CI 1.3, 6.1)) and boys (3.5 mmHg (95%CI 1.2, 5.8)). Diastolic BP and cholesterol concentrations were significantly positively (HDL cholesterol negatively) associated with high BMI and large WC, too. Normal weight children with a history of overweight did not have higher blood pressure levels or adverse cholesterol concentrations than children that were normal weight at both ages.

**Conclusion:**

A high BMI and large WC were associated with higher BP levels and adverse cholesterol concentrations. WC should be taken into account when examining cardiovascular risk factors in children.

## Introduction

Overweight during childhood increases the risk of cardiovascular diseases later in life [1]–[4]. This is probably due to the tracking of childhood and adolescent obesity into adulthood [5]. Besides that, already in children, overweight is associated with higher blood pressure (BP) levels and adverse cholesterol concentrations [6]–[12]. WC might more specifically reflect adiposity than BMI which reflects both lean and fat mass [13], and in addition, fat distribution may be more important for cardiovascular diseases. However, the question whether WC is more strongly associated with cardiovascular risk than BMI in children is still unresolved [6], [11], [12], [14]. Previous studies mainly focused on cross-sectional relations between BMI and cardiovascular risk factors, whereas the effect of persistence or remission of overweight on levels of cardiovascular risk factors remained under studied. We hypothesize that longer duration of overweight puts children at increased risk of overweight-related health outcomes, like in adults [15]. We examined the association between high BMI and large WC on one hand and BP and cholesterol concentrations on the other hand in 12-year-old children. We also compared the relative influence of BMI and WC in the associations with BP and cholesterol. Besides that, we also studied BP and cholesterol at age 12 years in children who lost, gained or maintained a high BMI or large WC between the ages of 8 and 12 years as compared to children with normal BMI or WC at both ages.

**Figure 1:**
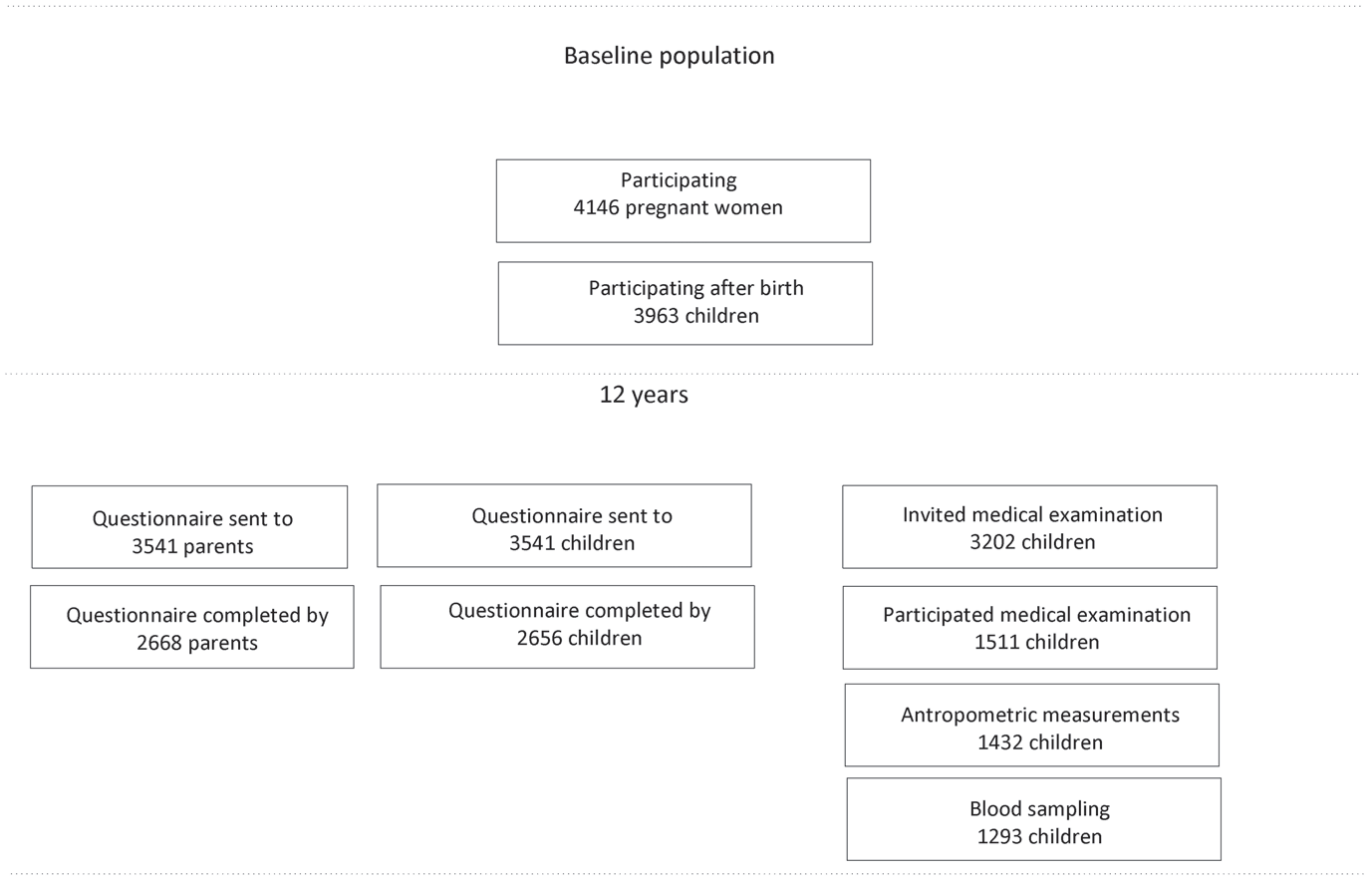
Flow chart.

## Methods

### Ethics Statement

This research was performed in accordance with the ethical principles for medical research involving human subjects outlined in the Declaration of Helsinki. Therefore, the study protocol was approved by the Medical Ethics Committees of the participating institutes (Rotterdam, start project MEC 132.636/1994/39 and 137.326/1994/130; Groningen, start project MEC 94/08/92; Utrecht, start project MEC-TNO oordeel 95/50; Utrecht 12 years 07–337/K). All parents and the children gave written informed consent.

### Study Design

The children in this study are participants of the Prevention and Incidence of Asthma and Mite Allergy (PIAMA) birth cohort study and were born in 1996–1997. A detailed description of the study design has been published previously [16]. The mothers were recruited from the general population during pregnancy visiting one of 52 prenatal clinics. Postal questionnaires were sent to the parents during pregnancy, at the child’s ages of 3 and 12 months, and yearly thereafter up to the age of 8 years. At the child’s age of 11 years the parents received a questionnaire and a separate questionnaire was sent to the children. A medical examination took place during a home visit at age 12 years. For the medical examination at the age of 8 years children were invited to the hospital, local health centers or a home visit. The study protocol was approved by the medical ethics committees of the participating institutes and all parents gave written informed consent.

**Table 1:** General characteristics of the study population, separately for girls and boys.

	Girls	n = 732	Boys		n = 700
	*Mean (sd)*		*Mean (sd)*		
Age (year)	12.7	(0.4)	12.7	(0.4)	
Weight (kg)	48.9	(9.4)	47.5	(9.2)	
Height (cm)	160.5	(7.2)	159.5	(8.2)	
Waist circumference (cm)	66.1	(6.5)	66.8	(6.8)	
BMI (kg/m^2^)	18.9	(2.7)	18.6	(2.6)	
Weight z-score	0.12	(1.0)	0.18	(1.0)	
Height z-score	0.21	(1.0)	0.13	(1.0)	
Weight for height z-score	−0.13	(1.0)	0.11	(1.1)	
BMI z-score	0.05	(1.1)	0.19	(1.1)	
WC z-score	0.26	(0.9)	0.08	(1.0)	
Birth weight (g)	3466	(505)	3600	(543)	
Systolic blood pressure (mmHg)	114	(9.5)	115	(8.9)	
Diastolic blood pressure (mmHg)	67	(6.3)	67	(6.6)	
Puberty development scale *	1.8	(0.6)	1.3	(0.3)	
Total cholesterol (mmol)	4.1	(0.6)	4.0	(0.7)	
HDL cholesterol (mmol)	1.4	(0.3)	1.4	(0.3)	
Total-to-HDL cholesterol ratio	3.1	(0.7)	3.1	(0.8)	
HbA1c (mmol/mol)	32	(2.4)	32	(2.2)	
	*n (%)*		*n (%)*		
Overweight (including obesity)	80	(11.0)	79	(11.3)	
Obesity	6	(0.8)	8	(1.2)	
BMI category 8 and 12 years					
Normal-normal	550	(88.0)	505	(87.8)	
High-normal	23	(3.7)	13	(2.3)	
Normal-high	16	(2.6)	27	(4.7)	
High-high	36	(5.8)	30	(5.2)	
WC category 8 and 12 years					
Normal-normal	538	(86.1)	494	(85.9)	
High-normal	29	(4.6)	21	(3.7)	
Normal-high	24	(3.8)	23	(4.0)	
High-high	34	(5.4)	37	(6.4)	
Systolic blood pressure >130 mmHg**	45	(6.2)	36	(5.2)	
Diastolic blood pressure >85 mmHg**	1	(0.1)	3	(0.4)	
HDL cholesterol <1.03 mmol**	85	(13.4)	88	(13.7)	
Maternal overweight before pregnancy	113	(15.5)	122	(17.5)	
Maternal education					
Low	122	(16.8)	120	(17.2)	
Intermediate	316	(43.6)	276	(39.6)	
High	287	(39.6)	301	(43.2)	
Maternal smoking	106	(14.6)	86	(12.3)	
Allergic mother ***	243	(33.4)	222	(31.9)	
Watching TV					
≤1 day/week	44	(6.2)	41	(6.0)	
2–4 days/week	103	(14.4)	81	(5.8)	
5–6 days/week	133	(18.6)	116	(17.1)	
daily	436	(60.9)	442	(65.0)	
Computer					
≤1 day/week	98	(13.7)	41	(6.0)	
2–4 days/week	278	(38.9)	195	(28.7)	
5–6 days/week	205	(28.6)	184	(27.1)	
daily	135	(18.9)	260	(38.2)	
Region ****					
North	255	(35.1)	204	(29.3)	
West	305	(42.0)	334	(47.9)	
Middle	167	(23.0)	159	(22.8)	

* The PDS ranges from 1 to 5 where a score of 1 indicates that puberty has not started yet and a score of 5 indicates that the puberty development seems complete. [19].

** The International Diabetes Federation provides thresholds for defining children at risk of future cardiovascular disease for HDL cholesterol and blood pressure. [28].

*** Allergic mother: ≥1 time yes to ‘ever asthma’, ‘allergy to pets’, ‘allergy to house dust mite’, or ‘nose allergy like hay fever’.

**** Regions: North: provinces Groningen, Friesland, Drenthe; West: region of Rotterdam; Middle: provinces Utrecht and Gelderland.

### Study Population

At baseline, the cohort consisted of 4146 pregnant women, 183 being lost to follow-up before any data of the child had been collected, thus the study started with 3963 newborns. At the age of 11 years 3541 children were still in the study and received a questionnaire. (Figure 1: Flow diagram) Questionnaires were returned by 2656 children and 2668 parents. For the medical examination, 3202 children were invited. 1511 children agreed to participate. Finally, the medical examination, including anthropometric measurements and BP measurements, was performed in 1432 children. Blood samples were taken from 1293 children, and cholesterol concentrations were available for 1285 children. Not everyone had all outcome measures available; this resulted in slightly different numbers of children in the analyses regarding BP and cholesterol concentrations. One child was excluded from the analyses with cholesterol because of very unlikely total and HDL cholesterol concentrations.

1215 children participating in the medical examination at 12 years also participated in the medical examination of 8-year-olds. This resulted in 1156 children for the analyses of BMI and WC at age 8 and 12 years with BP, and 994 children for the analyses with cholesterol concentrations.

**Table 2:** Associations between high BMI and large WC and systolic and diastolic blood pressure in 1375 12-year-old girls and boys.

Blood pressure	Girls n = 706				Boys n = 669			
	Systolic BP		Diastolic BP		Systolic BP		Diastolic BP	
	β	95% CI	β	95% CI	β	95% CI	β	95% CI
BMI 90^th^ centile								
Model B	**4.85**	**(2.49, 7.21)**	**2.44**	**(0.79, 4.09)**	**4.20**	**(1.94, 6.47)**	**3.39**	**(1.64, 5.13)**
Model C	**3.30**	**(0.70, 5.89)**	1.60	(−0.22, 3.42)	1.51	(−1.06, 4.07)	1.60	(−0.69, 3.58)
WC 90^th^ centile								
Model B	**3.71**	**(1.31, 6.12)**	**1.96**	**(0.28, 3.63)**	**3.50**	**(1.18, 5.81)**	**3.89**	**(2.12, 5.66)**
Model C	1.31	(−1.21, 3.83)	0.93	(−0.85, 2.71)	0.32	(−2.15, 2.79)	**2.98**	**(1.04, 4.92)**

BMI: body mass index, WC: waist circumference, BP: blood pressure.

Model B: adjusted for cuff size, pre-pregnancy maternal overweight, puberty development scale, age at the time of the measurements, height.

Model C: additionally adjusted for WC (in BMI analyses) and BMI (in WC analyses).

### Exposure Measures: Weight, Height and WC

The measurements during the medical examination were performed by trained research staff using calibrated measuring equipment. WC was measured midway between the lowest rib and the iliac crest. WC (cm) was measured twice and rounded at one decimal, the mean of the two waist measurements being used in the analyses. Weight was measured at the nearest 0.1 kg and height (cm) was measured at one decimal. All anthropometric variables were measured while the children were wearing underwear only. The same procedures were followed at ages 8 and 12 year. BMI was calculated as weight in kilograms divided by height squared in meters (kg/m^2^).

Z-scores of BMI and WC for age and gender were calculated by using the reference growth curves of the Dutch Fourth nationwide Growth Study [17]. We divided the children in two categories in our analyses, separately for WC and BMI, and for girls and boys, based on the distribution of the BMI z-score and the WC z-score; 1) above the 90^th^ percentile (high BMI, large WC) and 2) equal to or below the 90^th^ percentile, which we call ‘normal’. A ‘high’ BMI corresponds to a BMI z-score of 1.44 in girls and 1.55 in boys. A ‘large’ WC corresponds to a WC z-score of 1.46 in girls and 1.43 in boys. Overweight and obesity were defined according to standard international definitions (IOTF), specified for age and gender [18].

**Table 3:** Associations between high BMI and large WC and total and HDL cholesterol concentrations and total-to-HDL cholesterol ratio in 1168 12-year-old girls and boys.

Cholesterol	Girls n = 581						Boys n = 587					
	Total cholesterol		HDL cholesterol		Total-to-HDLcholesterol ratio		Total cholesterol		HDL cholesterol		Total-to-HDL cholesterol ratio	
	β	95% CI	β	95% CI	β	95% CI	β	95% CI	β	95% CI	β	95% CI
**BMI 90^th^ centile**												
Model B	0.03	(−0.14, 0.20)	−**0.16**	**(**−**0.24,** −**0.08)**	**0.49**	**(0.29, 0.69)**	**0.44**	**(0.26, 0.61)**	−**0.21**	**(**−**0.29,** −**0.13)**	**0.94**	**(0.73, 1.15)**
Model C	−0.13	(−0.32, 0.07)	−0.06	(−0.15, 0.03)	0.11	(−0.12, 0.33)	0.17	(−0.04, 0.38)	−0.07	(−0.17, 0.03)	**0.40**	**(0.14, 0.65)**
**WC 90^th^ centile**												
Model B	0.16	(−0.01, 0.33)	−**0.18**	**(**−**0.26,** −**0.10)**	**0.66**	**(0.46, 0.86)**	**0.63**	**(0.46, 0.81)**	−**0.19**	**(**−**0.27,** −**0.11)**	**1.06**	**(0.85, 1.28)**
Model C	0.11	(−0.08, 0.30)	−0.11	(−0.19, 0.02)	**0.44**	**(0.22, 0.66)**	**0.51**	**(0.31, 0.71)**	−0.07	(−0.16, 0.03)	**0.68**	**(0.44, 0.92)**

BMI: body mass index, WC: waist circumference, HDL: high density lipoprotein.

Model B: adjusted for pre-pregnancy maternal overweight, puberty development scale, age at the time of the measurements, height.

Model C: additionally adjusted for WC (in BMI analyses) and BMI (in WC analyses).

### Outcome Measures: BP and Cholesterol Concentrations

BP was measured using automatic BP meters (Omron M6 (Omron Healthcare Europe BV, Hoofddorp, the Netherlands). Cuff-sizes of either 15–22 (small) or 22–32 cm (normal) were used dependent on the mid-upper arm circumference. The cuff was placed at the non-dominant arm. Systolic and diastolic BP were measured at least two times with 5 minutes intervals according to the standard protocol while the child was seated and with the arm resting on their legs. We used the mean of the measures.

Serum total and HDL cholesterol concentrations were determined enzymatically using Roche automated clinical chemistry analyzers (Roche Diagnostics, Indianapolis). Additionally the ratio between total and HDL cholesterol was calculated (total-to-HDL cholesterol ratio).

### Potential Confounders

Potential confounders considered included the child’s height and exact age at the time of clinical examination, birth weight, maternal education (low, intermediate and high education), maternal overweight before pregnancy (BMI ≥25 kg/m^2^), maternal smoking during pregnancy, TV watching and computer time (times per week) reported in the child questionnaire around 11 years, and pubertal development scale (PDS) (assessed by child report [19]).

**Table 4:** Associations between high BMI and large WC of 8-and-12-year old children and systolic and diastolic blood pressure in 12-year-old girls and boys compared with girls and boys with a normal BMI or WC at both ages (normal-normal).

		Girls n = 606				Boys n = 550			
		Systolic BP		Diastolic BP		Systolic BP		Diastolic BP	
		β	95% CI	β	95% CI	β	95% CI	β	95% CI
BMI 90^th^ centile									
B	High-Normal	−2.71	(−6.81, 1.40)	−2.09	(−5.02, 0.84)	2.27	(−1.88, 6.43)	−2.27	(−5.47, 0.93)
	Normal-High	2.47	(−1.58, 6.53)	0.46	(−2.44, 3.35)	3.00	(−0.76, 6.75)	**3.76**	**(0.87, 6.65)**
	High-High	**5.42**	**(2.44, 8.40)**	**3.23**	**(1.10, 5.36)**	**4.86**	**(1.83, 7.89)**	**2.81**	**(0.48, 5.14)**
C	High-Normal	−2.80	(−7.38, 1.78)	−2.04	(−5.31, 1.23)	0.84	(−3.55, 5.22)	−**3.59**	**(**−**6.95,** −**0.23)**
	Normal-High	1.26	(−3.38, 5.89)	−0.16	(−3.47, 3.14)	2.34	(−1.71, 6.39)	2.46	(−0.65, 5.57)
	High-High	**4.82**	**(0.32, 9.32)**	3.01	(−0.20, 6.23)	1.42	(−2.71, 5.54)	−0.46	(−3.62, 2.71)
WC 90^th^ centile									
B	High-Normal	0.84	(−2.65, 4.32)	0.22	(−2.27, 2.70)	1.56	(−2.14, 5.27)	0.13	(−2.73, 2.99)
	Normal-High	**3.68**	**(0.06, 7.30)**	1.71	(−0.87, 4.29)	1.81	(−1.78, 5.43)	**3.31**	**(0.53, 6.10)**
	High-High	**4.21**	**(0.90, 7.52)**	**2.39**	**(0.03, 4.75)**	**6.53**	**(3.30, 9.76)**	**4.36**	**(1.87, 6.85)**
C	High-Normal	−0.11	(−4.25, 4.04)	−0.29	(−3.25, 2.67)	1.34	(−2.54, 5.22)	1.05	(−1.93, 4.03)
	Normal-High	2.43	(−1.72, 6.57)	1.27	(−1.69, 4.22)	0.85	(−3.08, 4.79)	2.64	(−0.38, 5.65)
	High-High	0.78	(−4.04, 5.61)	0.33	(−3.11, 3.77)	**5.47**	**(1.04, 9.90)**	**5.03**	**(1.63, 8.42)**

BMI: body mass index, WC, waist circumference, BP: blood pressure.

Model B: adjusted for cuff size, pre-pregnancy maternal overweight, puberty development scale, age at the time of the measurements, height.

Model C: additionally adjusted for WC (in BMI analyses) and BMI (in WC analyses).

### Statistical Analyses

General characteristics of the study population were calculated for boys and girls separately. First, we performed linear regression analyses with exposure (BMI and WC) and outcome (BP and cholesterol) measures around the age of 12 years. A potential confounder was included in the regression analyses when it changed the association between the exposure and outcome measures >10%. Three models were used in the regression analyses: Model A: adjusted for cuff size (only in BP analyses) and child’s age at time of the medical examination and Model B: additional adjustment for PDS, maternal pre-pregnancy overweight and the child’s height. In model C we adjusted the two exposure measures for each other to examine whether one the two measures explained the associations most strongly. Thus, model C was one model including BMI and WC and confounders. All analyses were stratified for gender. Because BMI and WC are highly correlated we performed a test of colinearity by determining the variance inflation factor for BMI and WC in model C. The variance inflation factors ranged from 1.6 to 2.0, suggesting no colinearity.

Second, linear regression analyses were performed with BMI and WC at age 8 and 12 years and outcome measures at age 12 years. For this purpose subgroups were created; children with a high BMI or large WC at both ages (‘high-high’), children with a high BMI or large WC at age 8 and a normal BMI or WC at age 12 years (‘high-normal’) and children with a normal BMI/WC at age 8 and a high BMI or large WC at age 12 years (‘normal-high’) were compared with children with a normal BMI or WC at both ages (‘normal-normal’). These analyses were equal to the analyses previously performed regarding stratification and adjustment. To study whether BMI or WC had a larger role in the associations with BP and cholesterol we used model C including both measures. We included the high-high, high-normal, and normal-high groups of BMI and of WC in one model, together with the confounders. All analyses were performed with SAS software version 9.2 (SAS Institute, Inc., Cary, NC).

To test whether BMI or WC was associated more strongly with BP and cholesterol concentrations, we used the following procedure to estimate confidence intervals for the difference in the associations of BMI and WC. 1000 bootstrap replications were performed to estimate mean differences between estimates for models with BMI and models with WC, and their confidence intervals [20]. The mean difference between the estimates of models with BMI and models with WC provides a test of heterogeneity between the estimates of BMI and WC.

**Table 5:** Associations between high BMI and large WC of 8-and-12-year-old children and total and HDL cholesterol concentrations and total-to HDL cholesterol ratio in 12-year-old girls and boys compared with girls and boys with a normal BMI or WC at both ages (normal-normal).

		Girls n = 505						Boys n = 489					
		Totalcholesterol		HDLcholesterol		Total-to-HDLcholesterol ratio		Totalcholesterol		HDLcholesterol		Total-to-HDLcholesterol ratio	
		β	95% CI	β	95% CI	B	95% CI	β	95% CI	β	95% CI	β	95% CI
BMI 90^th^ centile													
B													
	High-Normal	0.25	(−0.07, 0.57)	−0.04	(−0.19, 0.10)	0.29	(−0.09, 0.68)	0.13	(−0.18, 0.45)	0.08	(−0.07, 0.22)	0.01	(−0.38, 0.40)
	Normal-High	0.26	(−0.05, 0.57)	−0.09	(−0.24, 0.05)	**0.47**	**(0.10, 0.84)**	0.23	(−0.06, 0.52)	−**0.16**	**(**−**0.29,** −**0.03)**	**0.69**	**(0.33, 1.04)**
	High-High	−0.08	(−0.30, 0.14)	−**0.19**	**(**−**0.29,** −**0.09)**	**0.48**	**(0.22, 0.75)**	**0.53**	**(0.30, 0.77)**	−**0.19**	**(**−**0.30,** −**0.08)**	**0.98**	**(0.69, 1.27)**
C													
	High-Normal	0.06	(−0.30, 0.41)	−0.04	(−0.20, 0.13)	0.10	(−0.32, 0.53)	−0.04	(−0.36, 0.29)	0.13	(−0.02, 0.29)	−0.29	(−0.69, 0.11)
	Normal-High	−0.04	(−0.41, 0.33)	−0.05	(−0.21, 0.12)	0.07	(−0.37, 0.50)	−0.04	(−0.36, 0.27)	−0.13	(−0.28, 0.02)	0.38	(−0.00, 0.76)
	High-High	−0.40	(−0.75, 0.05)	−0.13	(−0.29, 0.03)	−0.00	(−0.41, 0.41)	0.03	(−0.38, 0.32)	−0.07	(−0.24, 0.10)	0.11	(−0.32, 0.54)
WC 90^th^ centile													
B													
	High-Normal	0.17	(−0.08, 0.40)	−0.03	(−0.14, 0.08)	0.21	(−0.07, 0.50)	0.09	(−0.19, 0.37)	−0.07	(−0.21, 0.06)	0.28	(−0.06, 0.62)
	Normal-High	**0.37**	**(0.10, 0.62)**	−0.11	(−0.23, 0.02)	**0.62**	**(0.30, 0.93)**	**0.58**	**(0.30, 0.86)**	−**0.13**	**(**−**0.26, 0.00)**	**0.84**	**(0.49, 1.18)**
	High-High	0.06	(−0.19, 0.29)	−**0.22**	**(**−**0.33,** −**0.10)**	**0.70**	**(0.40, 0.99)**	**0.71**	**(0.47, 0.95)**	−**0.20**	**(**−**0.32,** −**0.09)**	**1.22**	**(0.92, 1.52)**
C													
	High-Normal	0.30	(−0.00, 0.61)	0.03	(−0.11, 0.17)	0.19	(−0.17, 0.55)	0.10	(−0.19, 0.40)	−0.10	(−0.24, 0.04)	0.34	(−0.02, 0.70)
	Normal-High	**0.45**	**(0.13, 0.78)**	−0.07	(−0.21, 0.08)	**0.59**	**(0.21, 0.97)**	**0.60**	**(0.29, 0.92)**	−0.07	(−0.22, 0.08)	**0.68**	**(0.30, 1.07)**
	High-High	0.37	(−0.01, 0.74)	−0.12	(−0.29, 0.06)	**0.69**	**(0.24, 1.13)**	**0.74**	**(0.36, 1.11)**	−0.09	(−0.34, 0.01)	**1.16**	**(0.70, 1.61)**

BMI: body mass index, WC, waist circumference, HDL: high density lipoprotein.

Model B: adjusted for pre-pregnancy maternal overweight, puberty development scale, age at the time of the measurements, height.

Model C: additionally adjusted for WC (in BMI analyses) and BMI (in WC analyses).

## Results

The mean BMI z-score at 12 years of age was 0.05 in girls and 0.19 in boys and the mean WC z-score was 0.26 in girls and 0.08 in boys (Table 1). This means that on average, girls had similar BMI and larger WC compared with the reference population and, in contrast, that boys had higher BMI and similar WC than the reference population. Children who maintained their high BMI status had higher BMI at 12 years than children who changed from normal BMI at 8 years to high BMI at 12 years. For example, the mean BMI of girls in the ‘high-high’ BMI group was 25.4 kg/m^2^ compared with 23.9 kg/m^2^ in the ‘normal-high’ group, and in boys the mean BMI was 25.3 kg/m^2^ and 23.4 kg/m^2^ in the two groups respectively. Similar results were found with the WC groups. The mean (standard deviation (std)) systolic BP was 114 mmHg (9.5) in girls and 115 mmHg (8.9) in boys and the diastolic BP was 67 mmHg (6.3) in girls and 67 mmHg (6.6) in boys. All children with a high BMI were overweight according to the IOTF cut off points. A few children (18 boys and 29 girls) were overweight according to the IOTF cut off points, but did not have a BMI z-score above the 90^th^ percentile.

### BMI and WC in Relation to BP

In linear regression analyses we observed statistically significant associations between high BMI and large WC and BP. Systolic BP was 5.37 mmHg (95%CI 3.07, 7.66) higher in girls and 3.41 mmHg (95%CI 1.16, 5.68) higher in boys with a high BMI than in girls and boys with normal BMI (Model A). A large WC was associated with 4.30 mmHg (95%CI 1.95, 6.65) higher systolic BP in girls and 3.88 mmHg (95%CI 1.55, 6.20) higher systolic BP in boys (Model A). These associations changed somewhat after adjustment for confounders in model B (Table 2). After adjustment for the other exposure (BMI or WC) in model C all associations became weaker; only the associations between BMI and systolic BP in girls and WC and diastolic BP in boys remained significant (Table 2).

### BMI and WC in Relation to Cholesterol Concentrations

We found only significantly higher total cholesterol concentrations in boys with a high BMI (0.36 mmol (95%CI 0.19, 0.53)) and large WC (0.51 mmol (95%CI 0.34, 0.69)) (Model A), not in girls. HDL cholesterol concentrations were significantly lower in boys and girls with a high BMI and large WC after adjustment for confounders (Table 3). In line with these results, total-to-HDL cholesterol ratio was significantly higher in boys and girls with a high BMI or a large WC in adjusted analyses in Model B (Table 3). Similarly to the associations with BP, all associations between BMI and WC and cholesterol measures became weaker after inclusion of WC or BMI (Model C). The associations with WC seemed to attenuate less after adjustment for BMI than the associations with BMI after adjustment for WC.

### Prospective Analyses of BMI and WC at Ages 8 and 12 Years

Children with a high-high BMI status had statistically significantly higher BP levels at age 12 years than children with a normal-normal BMI status (Table 4). Similarly, children with a high-high WC status had statistically significantly higher BP levels than children with a normal-normal WC status (Table 4). Girls with a normal-high WC status had statistically significantly higher systolic BP and boys with a normal-high WC status had statistically significantly higher diastolic BP than children with a normal-normal WC status. Children with a high-normal BMI or WC status did not have higher BP at 12 years of age than children who had a normal-normal BMI or WC status. The analyses with BMI and WC in the same model showed that the associations between a high-high BMI status and BP attenuated less in girls whereas the associations between a high-high WC status and BP attenuated less in boys specifically.

Significantly higher total cholesterol concentrations (boys only) (0.53 mmol (95%CI 0.30, 0.77)), higher total/HDL cholesterol ratio (boys: 0.98 (95%CI 0.69, 1.27); girls: 0.48 (95%CI 0.22, 0.75)), and lower HDL cholesterol concentrations (boys: −0.19 mmol (95%CI −0.30, −0.08); girls: −0.19 (95%CI −0.29, −0.09)) were found with high-high BMI status in adjusted analyses (Model B) (Table 5). To a lesser extent and mainly in boys, these associations were also present in the normal-high group. Similar, but somewhat larger, estimates were found for the associations between high-high WC status and cholesterol outcomes. Children with a high-normal BMI or WC status did not have more adverse cholesterol concentrations at 12 years of age than children who had a normal-normal BMI or WC status. After mutual adjustment for BMI and WC, generally the associations with WC did hold, whereas the associations with BMI attenuated substantially.

Using the bootstrap replications, we estimated whether the estimates of BMI and WC with BP and cholesterol did statistically significantly differ from each other. The results of these analyses did not show statistically significant differences between BMI and WC in relation to BP and cholesterol.

## Discussion

First, we found statistically significant associations between a high BMI and systolic and diastolic BP, and total (boys only) and HDL cholesterol concentrations. We also found lower HDL cholesterol and higher systolic BP, diastolic BP and total cholesterol concentrations with increasing WC. Second, in our study the associations of BMI and WC with BP and cholesterol concentrations were similar. Third, our study suggests that normal weight children with a history of overweight are not at increased risk of higher BP levels and adverse cholesterol concentrations.

### Strengths and Limitations

We studied the associations between BMI and WC and blood pressure, and cholesterol concentrations in a large prospective birth cohort. We were able to study several outcome measures in relation to professionally measured weight, height and waist circumference. In addition, we included the child’s BMI and WC at age 8 years to examine whether persistence of high BMI or large WC is important in associations with cardiovascular risk factors. We did not have 8-year anthropometric measures of all children used in cross-sectional analyses, but the results of the cross-sectional linear regression analyses in the subgroup of children with measurements at both ages were similar to those of the whole group with data available at 12 years. The number of children in the HN and NH subgroups in the prospective analyses might have been too low to detect statistically significant differences, *but the results are consistently in the same direction.* The children in our study population had higher educated mothers (low 16.9%, moderate: 41.3%, high:32.4%) than in the original study cohort (low 23.5%, moderate: 41.6%, high:28.9%). However, in additional analyses these variables turned out to be no effect modifier, indicating that the association of BMI and WC with blood pressure and cholesterol concentrations is not different in children from high educated mothers compared with low educated mothers. Therefore, we assume that the generalisability of our results is not affected.

### Interpretation and Comparison with Previous Studies

In our study, a high BMI and a large WC were associated with higher systolic and diastolic BP, and higher total and lower HDL cholesterol concentrations. These results are in line with previous studies [6]–[11], [21], [22]. Several potential mechanisms explaining the association between excess weight and BP have been hypothesized: through an increase in the sympathetic nervous activity, insulin resistance, and arterial stiffness [23]. These factors all increase the cardiac output and systemic vascular resistance.

We observed similar associations of BMI and WC with BP and cholesterol concentrations. Only a few studies have examined both BMI and WC in relation to cardiovascular risk factors [6], [11], [12]. Although WC is, on theoretical grounds [13], hypothesized to be more important for cardiovascular risk this has not been confirmed so far in children. Two studies in the Avon Longitudinal Study of Parents and Children (ALSPAC) cohort study compared the magnitudes of the associations between BMI and WC, respectively, and cardiovascular risk factors [6], [11]. In the ALSPAC cohort study WC did not seem to be more strongly associated with BP and cholesterol than BMI [6], [11]. Maximova et al. observed similar effects of BMI and WC on changes in systolic BP in 12- and 13-year-old US children [12]. In younger children (age 8–10 years) no superior ability of WC to identify children with elevated systolic BP was shown over BMI [14]. Waist circumference has been suggested to be a better indicator of total fat mass, and reflects abdominal fat mass. Research in adults on the distribution of body fat has shown that a more central distribution of fat is associated with adverse outcomes [24].

It has been hypothesized that WC is a better marker of cardiovascular risk factors than BMI is, but this is not supported by literature on children, as none of the observational studies described above found differences in the associations of BMI and WC with cardiovascular outcomes. As long as the preference of BMI over WC or visa versa is not unambiguous, we suggest that studies investigating adiposity and cardiovascular risk factors study both BMI and WC.

In our study, children with a persistently high BMI or WC status were at increased risk of higher BP and more adverse cholesterol concentrations than children with a normal-high BMI or WC status. However, our study also suggests that normal weight children with a history of overweight are not at increased risk of higher BP levels and adverse cholesterol concentrations. Because of the small number of children in the HN and NH subgroups however, the results of these analyses should be interpreted with caution. Only two previous studies measured BMI at two time points [11], [25]. Lawlor et al. observed only in girls that a history of overweight did not put normal weight children at increased risk, boys who changed from overweight to normal weight showed risk factor profiles intermediate between those seen in boys who were normal weight at both ages or overweight at both ages [11]. Mamun et al. showed that children who were overweight at age 5 years and normal weight at age 14 years did have similar mean levels of BP compared with children who were normal weight at age 5 and age 14 years [11], [25]. Also in line with our results is their finding of higher BP levels in children who were overweight at both 5 and 14 years of age than BP in children who were normal weight at both ages. However, in contrast with our findings, Mamun et al. and Lawlor et al. showed higher sBP in children who were normal weight at 5 and 9–12 years respectively and overweight/obese at age 14 and 15–16 respectively than in children that were normal weight at both ages. We did show an increased risk of higher BP in these children, but our findings were not statistically significant. The different findings of the present study compared with the previous studies may be explained by the larger subgroups higher percentages of obese children in the previous studies. The results of the previous studies and ours that normal weight children with a history of overweight were not at increased risk of high blood pressure suggest that the high-risk status of overweight children for adult cardiovascular disease may be reversible. In recent studies on the associations between childhood obesity and adult cardiovascular disease, obese children were at increased risk of adult cardiovascular disease. However, when adult BMI was taken into account, only obese children who maintained their obesity into adulthood were at increased risk of cardiovascular disease. These results support the hypothesis that the high-risk status of overweight children for adult cardiovascular disease is reversible [26], [27].

### Concluding Remarks

This study focused on the relative importance of WC in the associations with BP and cholesterol. We showed similar associations of BMI and WC with BP and cholesterol concentrations. In our study, normal weight children with a history of overweight did not have higher BP levels or adverse cholesterol concentrations than children that were normal weight at both ages. This may imply that the effects of overweight on cardiovascular risk factors like BP and cholesterol are reversible, and that changing from overweight to normal weight status is worthwhile. The small numbers in the subgroups have to be taken into account in interpreting these findings. In addition, children who were overweight at both ages had higher BP levels and more adverse cholesterol concentrations than overweight 12-year-old children without a history of overweight. This implies that the duration of overweight contributes substantially to the risk of adverse levels of cardiovascular markers in overweight children. We did not study the changes in BP levels and cholesterol concentrations with regard to the results on history of overweight. Many childhood overweight reduction interventions have been performed so far, and it might be interesting to monitor also cardiovascular risk factors in these intervention groups.
